# Application of empirical likelihood ratio test in AR(1) time series model for PM_2.5_ forecasting in Guwahati city of Assam

**DOI:** 10.1038/s41598-025-24076-7

**Published:** 2025-11-17

**Authors:** Vinitha Serrao, Satyanarayana Poojari, Ismail B., K. Aruna Rao

**Affiliations:** 1 Department of Applied Statistics and Data Science, Prasanna School of Public Health, Manipal Academy of Higher Education, Manipal, Karnataka 576104 India; 2 Department of Statistics, Yenepoya (Deemed to be University), Deralakatte, Mangalore, 574153 India; 3 Department of Statistics, Mangalore University, Mangalagangothri, Konaje, Karnataka 574199 India

**Keywords:** Climate sciences, Environmental sciences, Mathematics and computing

## Abstract

The Autoregressive model plays a vital role in time series analysis, as it efficiently captures short-term dependencies while maintaining simplicity. Accurate identification of the order of autoregressive models is essential for enhanced model performance and reliable forecasting. Traditional method of identifying AR (1) models relying on autocorrelation and partial autocorrelation plots, along with the Ljung-Box (LB) test, often suffer from subjectivity and potential overfitting. To overcome this limitation, this study proposes an Empirical Likelihood Ratio Test (ELRT) for assessing the suitability of AR (1) model in time series analysis. Through Monte Carlo simulation, performance of the ELRT is compared with the LB test in terms of empirical size and power. Simulation results indicate that the ELRT maintains empirical size more accurately while exhibiting superior power compared to LB test. The proposed test is further validated using major air pollutant, PM_2.5_ data from Guwahati, Assam. The empirical results show that the forecasted PM_2.5_ levels remain mostly in the “Poor” to “Very Poor” range during the initial months, indicating unhealthy air quality. A slight improvement to ‘Moderately Polluted’ is observed in the fourth month. The proposed approach offers a robust alternative for AR (1) model identification and improves the reliability of forecasts in air quality studies.

## Introduction

Time series analysis is a specialized field of data analysis that focuses on identifying patterns and trends in sequential data over time. Modelling and forecasting time series data are essential for informed decision-making and planning in various domains, such as air quality monitoring, meteorological prediction, finance, economics, and many other fields. Among the various times series models, the Autoregressive Integrated Moving Average (ARIMA) model introduced by Box and Jenkins^1^ is a commonly used method for analysing and forecasting time series data that exhibit non-stationarity. The ARIMA model is represented as ARIMA (p, d, q), where *p* indicates the number of autoregressive terms, $d$ reflects the degree of differencing required to achieve stationarity, and *q* denotes the number of moving average terms. By combining both autoregressive (AR) and moving average(MA) elements, the ARIMA model efficiently captures the inherent trends and patterns in time series data, enabling accurate predictions.

The AR models are effective in capturing short-term dependencies by predicting future values based on prior observations. The AR model is a fundamental tool in time series analysis, providing a comprehensive framework for identification, estimation, and diagnostic checking. The simplicity of AR models, combined with their capability to model temporal dependencies, makes them widely applicable. A simpler version of the AR model is the AR (1) model, which focuses exclusively on autoregressive dynamics of order 1.

Various estimation methods have been proposed to estimate parameters of AR (1) models, aiming to reduce bias and improve the precision of parameter estimates. One of the widely used techniques is the closed-form method known as the ${r}_{1}$ estimator introduced by Yule^2^ and later refined by Walker^3^ and Box and Jenkins^4^. Young^5^ introduced the C-statistic, a method for estimating parameters in AR models. This study introduces an alternative Yule-Walker equations method, a form of Ordinary Least Squares (OLS) estimation particularly for small sample sizes and helps improve parameter estimation and forecasting accuracy. Additionally, Maximum Likelihood Estimation an iterative technique has been introduced for estimation of AR model along with Bayesian Markov Chain Monte Carlo Several simulation studies^6^^,^^7^^,^^8^^,^^9^ have been conducted to compare the performance of closed-form methods, particularly the ${r}_{1}$ estimator, C-statistic, and OLS. The results suggest that when the length of time series data is short (e.g., length ≤50 observations), the estimates may be biased and show significant variability.

Philips and Han’s^10^ research marks a notable improvement in Gaussian inference for AR (1) models, presenting a unified approach that applies to both stationary and non-stationary cases. This method enhances the accuracy and consistency of parameter estimates, ensuring robust statistical inference across various autoregressive parameters. Hosein and Yeganegi^11^ highlight the critical role of selecting the appropriate lag order (H) when using the Ljung-Box test in time series analysis, as incorrect lag values may distort autocorrelation results. Arltova et al.^12^ suggest that the Augmented Dickey-Fuller test is better for shorter time series, while Phillips-Perron or Ng-Perron tests are more suitable for longer series, especially when the AR (1) parameter nears the unit root. These studies enhance the understanding of efficient inference techniques and test selection for AR (1) models.

There is a lack of comprehensive comparison between closed-form and iterative methods, leading to uncertainty about their relative performance in terms of bias and variability. Additionally, the robustness of these methods under model misspecification remains underexplored. Understanding their comparative performance and robustness is crucial for enhancing time series research and selecting methods that minimize bias, reduce variability, and ensure greater robustness in estimating empirical AR (1) models. Identifying the order of an AR series, particularly AR (1), is crucial for accurate time series analysis, as it impacts both estimation and forecasting. Accurate identification of the model’s order also avoids overfitting and underfitting.

The empirical likelihood (EL) method, first introduced by Owen in^13^ and^14^, has gained significant popularity for statistical inference due to its flexibility in constructing confidence regions that accurately capture the structure of the underlying distribution without assuming symmetry. EL approach has been extended to various applications, such as linear regression^15^, generalized linear models^16,17^. Later, Zhang et al.^18^ examined the robustness of EL for estimating integer-valued autoregressive (INAR) processes parameters. Extensions of the EL method have also been applied to time series models^19^. Ding and Wang^20^ further advanced the EL method by applying it to inference in INAR models. More recently, Satyanarayana et al.^21^ introduced an empirical likelihood ratio test for detecting AR (1) in regression model, demonstrating superior performance in both empirical size and power. Additionally, Zhang et al.^22^ proposed EL based test for INAR (1) models and derived the test statistic under null hypothesis. This approach provides unified inference for both stable and unstable processes.

As a real-life application, this study focuses on forecasting one of the most critical air pollutants i.e. PM_2.5_. Air pollution poses a major global challenge, significantly impacting human health, the environment, and the economy. Fine particulate matter (PM_2.5_), primarily emitted from transportation, industrial, and agricultural sources, presents serious health risks due to its ability to penetrate deep into the lungs and enter the bloodstream^23^. It is strongly associated with respiratory and cardiovascular diseases, lung cancer, and other chronic health conditions^24,25^. PM_2.5_ exposure can interfere with vital cellular processes such as autophagy, apoptosis, necrosis, and ferroptosis, contributing to tissue damage and disease progression^26^. Experimental studies further reveal that PM_2.5_ adversely affects kidney and epithelial cells by inducing epithelial-mesenchymal transition and altering the function of organelles^27^^,^^28^^,^^29^.

Climate change is expected to intensify exposure and worsen health outcomes, especially cardiovascular diseases^30–32^. Projections suggest mortality from PM_2.5_ could significantly decline under low-emission scenarios by 2100^33^. As PM_2.5_ pollution is common and long-lasting, there is a strong need for better air quality control and public health measures to reduce exposure. Accurate forecasting of PM_2.5_ helps to reduce the health and economic burden associated with pollution, making it a crucial tool for sustainable public health and environmental management. Recent studies in environmental monitoring and forecasting highlight the increasing complexity of datasets and the corresponding need for rigorous model adequacy testing. Pragasan and Ganesan^34^ conducted an assessment of air pollutants and identified pollution-tolerant vegetation for greenbelt development, highlighting the need for accurate identification of the appropriate time series model facilitates precise pollutant prediction, enabling timely and targeted greenbelt planning. Li et al.^35^ examined the interaction effects of PM_2.5_ and O₃ on respiratory health, underscoring the critical importance of accurate pollutant forecasting for PM_2.5_ public health interventions. Rabbani et al.^36^ applied machine learning, remote sensing, and chemometrics to assess environmental stress from air pollutants. This paper demonstrates robust pattern extraction from complex, high-dimensional data and highlights the diversity of modeling frameworks in air pollution research. Gu et al.^37^ proposed a Self-Adaptive Multiscale Transform Domain framework to integrate local and global information for improved air pollution monitoring, demonstrating how advanced data fusion methods can enhance spatio-temporal resolution in environmental datasets.

These studies highlight the increasing complexity of environmental datasets and the critical role of accurate forecasting for timely public health and mitigation policies. Consequently, there is a pressing need to correctly identify the order or suitable time series model to ensure reliable pollutant predictions. Robust model identification enhances forecast accuracy, thereby supporting effective and timely policy- making for environmental management.

The main objective of this study is to develop and evaluate an ELRT designed to assess the adequacy of an AR (1) time series model. The ELRT produces a data-driven test statistic that directly targets departures from the AR (1) hypothesis, rather than testing for generic form of autocorrelation. Methodologically, the ELRT differs from the Ljung–Box test by not relying on strict parametric distributional assumptions. Instead, it utilizes a nonparametric empirical likelihood framework, which enhances robustness and maintains validity across a wide range of real-world applications. In contrast, the widely used LB test is an omnibus portmanteau test, assessing overall serial correlation up to a chosen lag and often requiring subjective interpretation of ACF and PACF diagnostics. This limitation highlight the importance of need for a formal statistical test for order detection offering a more objective and dependable approach for identification compared to relying solely on visual plots. Under mild regularity conditions such as stationarity and standard moment assumptions, under the null hypothesis ELRT statistic follows a known asymptotic distribution. Simulation results indicate that the ELRT maintains nominal Type I error rates and achieves higher statistical power than the LB test in detecting subtle departures from the AR(1) structure, making it an appropriate choice for identifying AR (1) time series model. The paper is organized into five sections, with Section 2 outlining the proposed test. Section 3 compares the estimated type I error rate and power of the ELRT and LB tests using finite samples. Section 4 discusses a real-world example, with the paper’s conclusion presented in Section 5.

## Methods

### Autoregressive (AR) model

A time series $\left\{{X}_{t} ;t\in I\right\}$ that follows an $AR(p)$ process can be represented as:

1 $${X}_{t}={\phi }_{1}{X}_{t-1}+{\phi }_{2}{X}_{t-2}+\dots +{\phi }_{p}{X}_{t-p}+{\varepsilon }_{t}$$

where $\left\{{\varepsilon }_{t} ;t\in I\right\}$ a white noise process with mean 0 and variance ${\sigma }^{2}$. This model is denoted as ${X}_{t}\sim AR(p)$, where ${X}_{t}$ represents the current observation, ${X}_{t-1},{X}_{t-2},\dots ,{X}_{t-p}$ are the observations at the previous $p$ time points, ${\phi }_{1},{\phi }_{2},\dots ,{\phi }_{p}$ are the autoregressive coefficients, and ${\varepsilon }_{t}$ is the error term. For the AR (1) process, the model simplifies to:

2 $${X}_{t}={\phi }_{1}{X}_{t-1}+{\varepsilon }_{t },       t=\text{2,3},\dots n$$

where ${\phi }_{1}$ represents the autocorrelation coefficient, ${\varepsilon }_{t}$ are assumed to be serially independent and independent of ${X}_{t-1}$ for all $t,$ with mean 0 and variance ${{\sigma }_{\varepsilon }}^{2}$. The mean of AR (1) model i.e $E\left({X}_{t}\right)=0$ and $V({X}_{t})= \frac{{{\sigma }_{\varepsilon }}^{2}}{1-{\rho }^{2}}$. The autocorrelation between observations h time periods apart is

3 $${\rho }_{h}=\frac{cov({X}_{t},{X}_{t+h})}{V\left({X}_{t}\right)}$$

4 $${\rho }_{h}={{\phi }_{1}}^{h}$$

The ACF shows a specific pattern for autocorrelations. When ${\phi }_{1}$ is positive, as the lag (h) increases, ACF gradually decreases to zero. If ${\phi }_{1}$ is negative, the ACF also decays exponentially to zero, but the signs of the autocorrelations alternate between positive and negative. On the other hand, PACF clearly shows a sharp drop after lag 1, signifying that the AR process has an order of 1.

### Ljung –Box (LB) test

The LB test is a statistical method designed to check whether the autocorrelations in a time series differ significantly from zero. Unlike tests that assess randomness at single lags, this test evaluates multiple lags simultaneously, making it a comprehensive or portmanteau test.

The hypotheses for this test are defined as follows:

- ${H}_{o}:$ The residuals are uncorrelated, indicating the absence of autocorrelation.
- ${H}_{1}:$ The residuals are correlated, suggesting the presence of significant autocorrelation.

The test statistic is calculated using the formula

5 $$Q=n(n+2)\sum\nolimits_{k=1}^{h}\frac{{{\widehat{\rho }}_{k}}^{2}}{n-k}$$

where $n$ represents the sample size, ${\widehat{\rho }}_{k}$ denotes the sample autocorrelation at lag $k$, and $h$ indicates the number of lags under consideration. Under the null hypothesis, the Q statistic follows an asymptotic chi-squared distribution with *h* degrees of freedom.

### Proposed empirical likelihood ratio test for autoregressive of order 1

The ELRT is proposed to assess the adequacy of the AR(1) time series model as a nonparametric approach. The term “*non-parametric”* refers to the empirical likelihood approach employed in developing the ELRT statistic. The EL method combines the reliability of nonparametric methods with the effectiveness of the likelihood approach. Unlike traditional parametric methods, empirical likelihood does not assume a specific distribution for the data^13,14^. Instead, empirical likelihood is constructed based on moment conditions derived from the AR (1) model. This data-driven framework offers greater flexibility and robustness, as it allows for valid inference without imposing any specific distributional assumptions. This makes it especially effective in detecting subtle departures from the AR(1) structure in complex real-world data. The methodological development of the ELRT for the AR(1) time series model is outlined as follows.

Let ${x}_{1}, {x}_{2},. . . {x}_{n}$ are the samples from a distribution with continuous cumulative distribution function. Let ${p}_{i}$ denote the probability attached to ${x}_{i}$.The hypothesis of interest is ${H}_{0}$ : ${\phi }_{1} = 0$ against ${H}_{1}$ : ${\phi }_{1}> 0$, where $\rho$ denotes the autocorrelation coefficient. Under ${H}_{0}$, the ELRT statistic is given by

$$R\left(\phi \right) = {max}_{{p}_{2}, {p}_{3}\dots {p}_{n}}\left\{{\prod_{i=2}^{n}(n-1)}p_{i} | {p}_{i}\ge 0, \sum_{i=2}^{n}{p}_{i} = 1 , \sum_{i=2}^{n}{p}_{i}({x}_{i}-\overline{x })({x}_{i-1}-\overline{x })= 0 \right\}            (6)                                                                           $$

#### Theorem:

Under regularity conditions the asymptotic null distribution of $-2\text{log R}\left(\phi \right)$ is central chi-square with 1 degree of freedom.

#### Proof:

The Lagrange multiplier technique is used to solve this optimization problem.

Let $$G =\sum_{t=2}^{n}{\mathit{log}p}_{t} + \gamma \{{\sum }_{t=2}^{n}{p}_{t}-1 \}+\lambda \{\sum_{t=2}^{n}{p}_{t}({x}_{t}-\overline{x })({x}_{t-1}-\overline{x })- 0\}$$

$$\text{Equating }\frac{\delta G}{ \delta {p}_{t}}=0,\text{ leads to }{p}_{t}={\left[-\gamma -\lambda ({x}_{t}-\overline{x })({x}_{t-1}-\overline{x })\right]}^{-1}$$

$$\sum_{t=2}^{n}{p}_{t}\frac{\delta G}{\delta {p}_{t}}=0, gives \gamma =-n$$

$$Therfore, \quad{p}_{t}={\left[n-\lambda ({x}_{t}-\overline{x })({x}_{t-1}-\overline{x })\right]}^{-1}$$

$$where\: \lambda\: is\: the\: unique\: solution\: of\: \sum_{\text{t}=2}^{n}\frac{({x}_{t}-\overline{x })({x}_{t-1}-\overline{x })}{[n-\lambda ({x}_{t}-\overline{x })({x}_{t-1}-\overline{x })]}=0$$

$$Using\: Taylor \:expansion \:of\: \sum_{\text{t}=2}^{n}\frac{({x}_{t}-\overline{x })({x}_{t-1}-\overline{x })}{[n-\lambda ({x}_{t}-\overline{x })({x}_{t-1}-\overline{x })]} \:around\: \lambda\: =0\:\text{ results in}$$

$$\lambda =- \frac{\sum_{t=2}^{n}({x}_{t}-\overline{x })({x}_{t-1}-\overline{x })}{\frac{1}{(n-1)}{\sum_{t=2}^{n}[({x}_{t}-\overline{x })({x}_{t-1}-\overline{x })]}^{2}}$$

Using the Taylor expansion of $\mathit{log}\left(1-x\right)$, omitting the term $(n-1)log (n-1)$ which eventually gets cancelled,

$$\begin{aligned} & -2\sum_{\text{t}=2}^{\text{n}}\text{log}{p}_{t}=2\sum_{t=2}^{n }log\left[1-\frac{\lambda (({x}_{t}-\overline{x })({x}_{t-1}-\overline{x })) }{\left( n-1\right)}\right]\\ & =-2\frac{\lambda }{\left(n-1\right)}\sum_{t=2}^{n}({x}_{t}-\overline{x })({x}_{t-1}-\overline{x })-\frac{{\lambda }^{2}}{({n-1)}^{2}}{\sum_{t=2}^{n}[({x}_{t}-\overline{x })({x}_{t-1}-\overline{x })]}^{2}\\&= \frac{{[\sum_{t=2}^{n}({x}_{t}-\overline{x })({x}_{t-1}-\overline{x })]}^{2}}{\frac{1}{(n-1)} {\sum_{t=2}^{n}[({x}_{t}-\overline{x })({x}_{t-1}-\overline{x })]}^{2}}\end{aligned}$$

By LLN $\frac{1}{(\text{n}-1)}{\sum_{t=2}^{n}[({x}_{t}-\overline{x })({x}_{t-1}-\overline{x })]}^{2}\longrightarrow$ Var $(({x}_{t}-\overline{x })({x}_{t-1}-\overline{x }))$

7 $$\text{Therefore }-2\sum\nolimits_{\text{t}=2}^{\text{n}}\text{log}{p}_{t}=\frac{{[\sum_{t=2}^{n}({x}_{t}-\overline{x })({x}_{t-1}-\overline{x })]}^{2}}{\text{Var }(({x}_{t}-\overline{x })({x}_{t-1}-\overline{x }))}$$

Thus, test statistic, -$2log R\left(\phi \right)$ is asymptotically distributed as central ${\chi }^{2}$ with 1 degree of freedom. The ELRT is robust to distributional assumptions, while the performance of the LB test is heavily dependent on the normality assumption, which may not always be valid. The LB test is commonly used to confirm whether a series follows an AR (1) model, but its accuracy is compromised when the normality assumption is not met. In such cases, the nonparametric ELRT offers a more reliable alternative for testing the AR (1) structure.

The proposed ELRT offers several advantages over the traditional LB test. Unlike LB test which relies on subjective interpretation of ACF and PACF plots, the ELRT provides a data-driven framework for evaluating the suitability of an AR(1) model. While the LB test assesses general autocorrelation without reference to a specific model, the ELRT is explicitly formulated to test the AR (1) hypothesis. The key assumption conditions under which the ELRT performs optimally include (a) correct specification of the moment conditions associated with the AR(1) process, (b) stationarity of the time series, (c) independence and identical distribution of the error terms with finite variance, and (d) absence of model misspecification. The empirical likelihood framework also assumes the existence of finite higher-order moments, which supports the asymptotic properties of the test under the Wilks theorem.

Although ELRT have been developed to detect the presence of autocorrelation, specifically AR(1) error structures in regression models^21^ and INAR processes, the focus of the present study is distinctly different. The proposed ELRT is specifically designed to assess whether the observed time series itself follows an AR (1) model, rather than testing for autocorrelation in regression residuals. Unlike previous approaches, the moment conditions in this ELRT are derived directly from the time series process, not from regression errors. This distinction highlights the novelty of the proposed method in the context of time series model validation.

## Results

### Simulation study

A Monte Carlo simulation study was conducted to assess the finite sample performance of the proposed ELRT in detecting the suitability of AR(1) time series model. The main objective of the simulation study was to compare the empirical size and power of the ELRT with the traditional Ljung-Box (LB) test under varying autocorrelation level and sample size.

#### Data generation process

The following autoregressive model of order 1 was used for data generation:

$${X}_{t}= {{\phi }_{1} X}_{t-1} + {\varepsilon }_{t}, \text{t} =1,2,\ldots,\text{n}\: \text{where}\: X_t \sim AR(1).$$

Where ${\varepsilon }_{t}$ are independent and identically distributed (i.i.d.) normal errors with mean zero and finite variance and ${X}_{t}$ follows a stationary AR (1) process. Data were simulated using the *arima.sim*() function in the R programming environment, which is specifically designed for generating time series under ARIMA-type models. The autocorrelation parameter ${\phi }_{1}$ was varied across 10 values in the range [0,0.9] with increments of 0.1. This allowed evaluation of the test ability to detect serial dependence of varying strength. To evaluate finite sample performance, simulations were performed under multiple sample sizes combination : 30, 50, 80, 100, and 200. For each scenario, 5000 replications were generated and average values over 5000 replications are reported. The test are carried at a nominal significance level of 0.05.

#### Implementation of ELRT

A detailed R code is written for computing ELRT test statistics using *Rsolnp* package based on moment constraints derived from the AR(1) structure. This statistic asymptotically follows a chi-square distribution with 1 degree of freedom under the null hypothesis. For each simulated dataset, the ELRT statistic was calculated. If the statistic exceeded the critical value from the chi-square distribution with 1 degree of freedom at the 0.05 significance level (i.e., 3.84), the null hypothesis of no serial correlation was rejected.

#### Computation of empirical size and power

##### Empirical size

To compute the empirical size, data were generated under the null hypothesis with ${\phi }_{1}$=0. Both the ELRT and the LB tests were applied, and the proportion of times the null hypothesis was incorrectly rejected over 5000 replications was recorded as the empirical size for each sample size.

##### Empirical power

To assess power, data were generated from an AR(1) time series model with varying levels of autocorrelation by changing the value of ${\phi }_{1}$. The proportion of times the null hypothesis was rejected across 5000 replications was recorded as the empirical power of the test to detect autocorrelation. Table 1 presents the empirical size results of the ELRT and the LB test across different sample sizes under the assumption of normally distributed errors.

**Table 1:** Comparison of ELRT and LB test based on empirical size.

**Sample size**	**Test**	**Empirical size**
20	ELRT	0.083
	LB	0.02
30	ELRT	0.076
	LB	0.039
50	**ELRT**	**0.055**
	LB	0.033
80	ELRT	0.048
	LB	0.042
100	**ELRT**	**0.058**
	LB	0.034
200	ELRT	0.054
	**LB**	**0.046**

From the simulation results, several important implications were drawn. For a sample size of 20, the ELRT test tends to be slightly liberal, exceeding the nominal size, while the LB test is notably conservative. Neither test maintains the nominal level with sample sizes as small as 30. However, for sample sizes of 50 and above, the ELRT test consistently adheres to the nominal size, and the LB test closely approximates the fixed nominal level. Given that a minimum of 50 observations^1^ is recommended for reliable time series modeling, it is noteworthy that the ELRT test effectively maintains the nominal size from this threshold onward. In summary, the ELRT test consistently maintains the nominal size more reliably than the LB test.

**Fig. 1:**
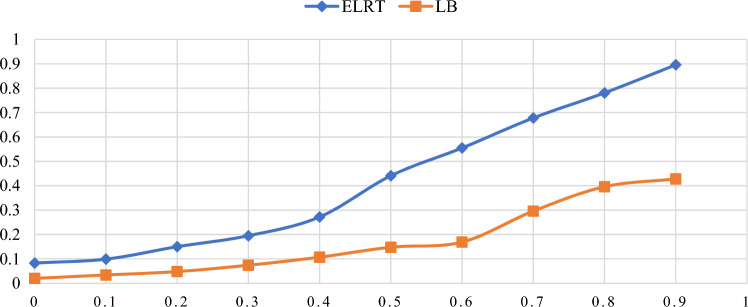
Rejection rates for ELRT and LB test when n= 20.

**Fig. 2:**
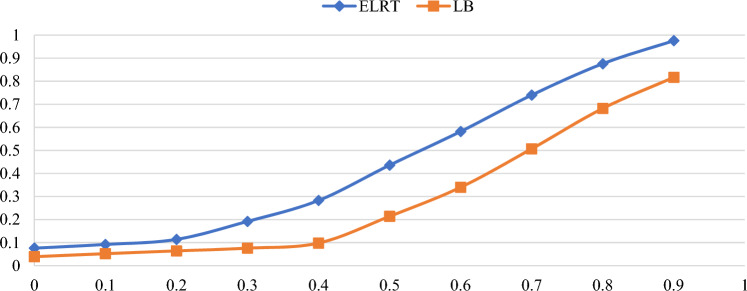
Rejection rates for ELRT and LB test when n= 30.

**Fig. 3:**
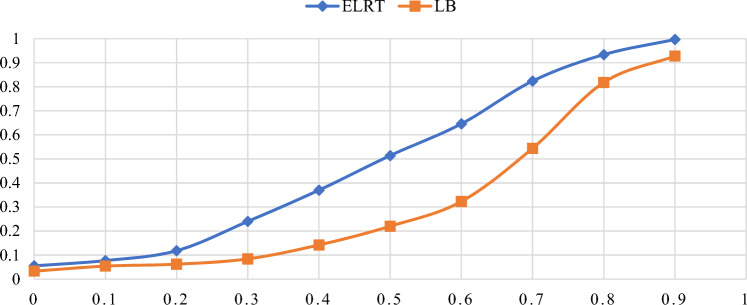
Rejection rates for ELRT and LB test when n= 50.

**Fig. 4:**
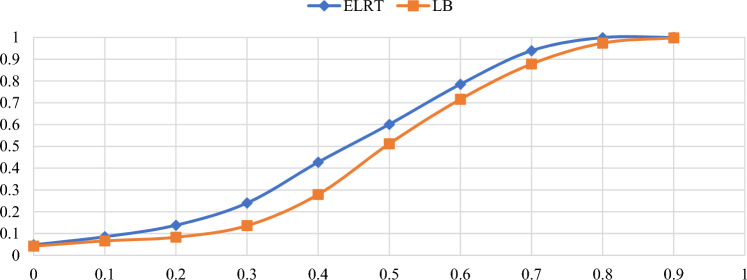
Rejection rates for ELRT and LB test when n= 80.

**Fig. 5:**
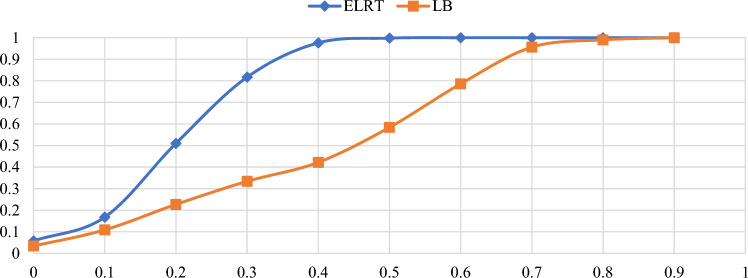
Rejection rates for ELRT and LB test when n= 100.

**Fig. 6:**
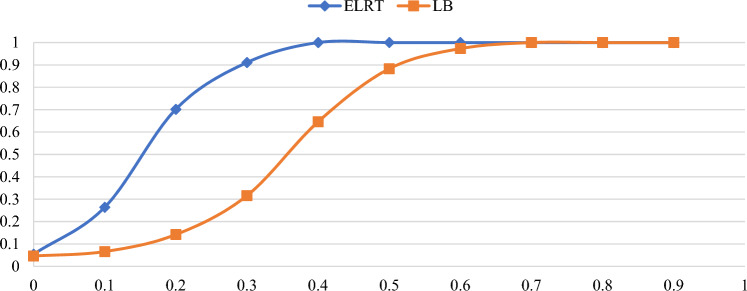
Rejection rates for ELRT and LB test when n= 200.

Figures 1,2,3,4,5,6 illustrate the empirical rejection rates across six different sample sizes. The vertical axis represents the rejection rate, while the horizontal axis displays varying values of the autocorrelation coefficient. Notably, the empirical power of the ELRT test surpasses that of the LB test, particularly with smaller sample sizes. For example, at a sample size of 20, the ELRT test shows a rejection rate of 0.896 for ${\phi }_{1}=0.9$, whereas the LB test’s rejection rate is 0.428. Furthermore, as the sample size increases, the ELRT consistently maintains higher power than the LB test, regardless of the autocorrelation level. Overall, in all scenarios, the ELRT test demonstrates superior performance compared to the LB test.

### Real life application

The Railway Colony station in Guwahati, Assam, is among the most polluted air quality stations in the city. For this study, monthly data on particulate matter 2.5(PM_2.5_) concentration data (measured in µg/m3) from the Railway Colony monitoring station, sourced from the Central Pollution Control Board (CPCB) of India, covering the period from 2022 to 2024. For the data analysis, the original monthly data was aggregated into weekly data. As part of the preprocessing, outlier analysis was conducted using box plots, which indicated the absence of significant outliers in the data. The dataset was also checked for missing observations, and the results indicated that approximately 2% of the data was missing. These missing values were imputed using the Last Observation Carried Forward (LOCF) method. Due to their small size, these particles can easily be inhaled, reaching deep into the lungs and potentially entering the bloodstream. This exposure can lead to serious health problems, including respiratory and heart diseases, as well as an increased risk of cancer. Longterm exposure to high concentrations of PM_2.5_ is associated with an elevated risk of premature death from these conditions. Given the severe health implications of fine particulate matter, the monitoring and forecasting of PM_2.5_ levels are essential for evidence-based environmental policymaking.

**Fig. 7:**
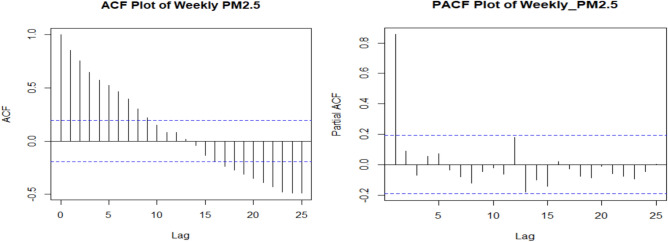
ACF and PACF plot of weekly PM_2.5_ level.

Based on the analysis, the estimated autocorrelation coefficient is 0.856. In the Figure 7, the PACF plot reveals a significant spike at lag 1, suggests that the series exhibits a first-order autoregressive structure. Additionally, the ACF plot given in figure 7 shows an exponential decay, which is a typical characteristic of autoregressive models. Further, this pattern in the ACF supports the conclusion that the data follows an AR (1) process. Therefore, the combination of the PACF and ACF patterns provides strong evidence for modeling the series using an AR (1) model. Following order selection, the AR(1) model was estimated using the method of maximum likelihood. To further evaluate the suitability of the AR(1) model, the LB test and ELRT were subsequently applied and results are provided in table 2.

**Table 2:** Results of ELRT and LB test for PM_2.5_ Level data.

Test	Test statistic	p value
Ljung -Box	2.68	0.1016
**ELRT**	**118.8332**	**<0.001**

The AR (1) model is fitted to the data, and the residuals are tested using the LB test. The Table 2 represents the results of ELRT and Ljung Box test for PM_2.5_ Level data. Since the p-value of LB test is greater than 0.05, suggests insignificant autocorrelation among the residuals, implying that the AR (1) model provides an adequate fit for the data.The ELRT of original series yielded a test statistic of 118.8332 with a p-value < 0.001, indicating significant autocorrelation at lag 1. These results imply that both LB test and ELRT identified significant autocorrelation of order 1, supporting the suitability of an AR (1) model. Therefore, the AR (1) model is fitted to obtain forecasts for the next 28 weeks (January to April 2025).

**Fig. 8:**
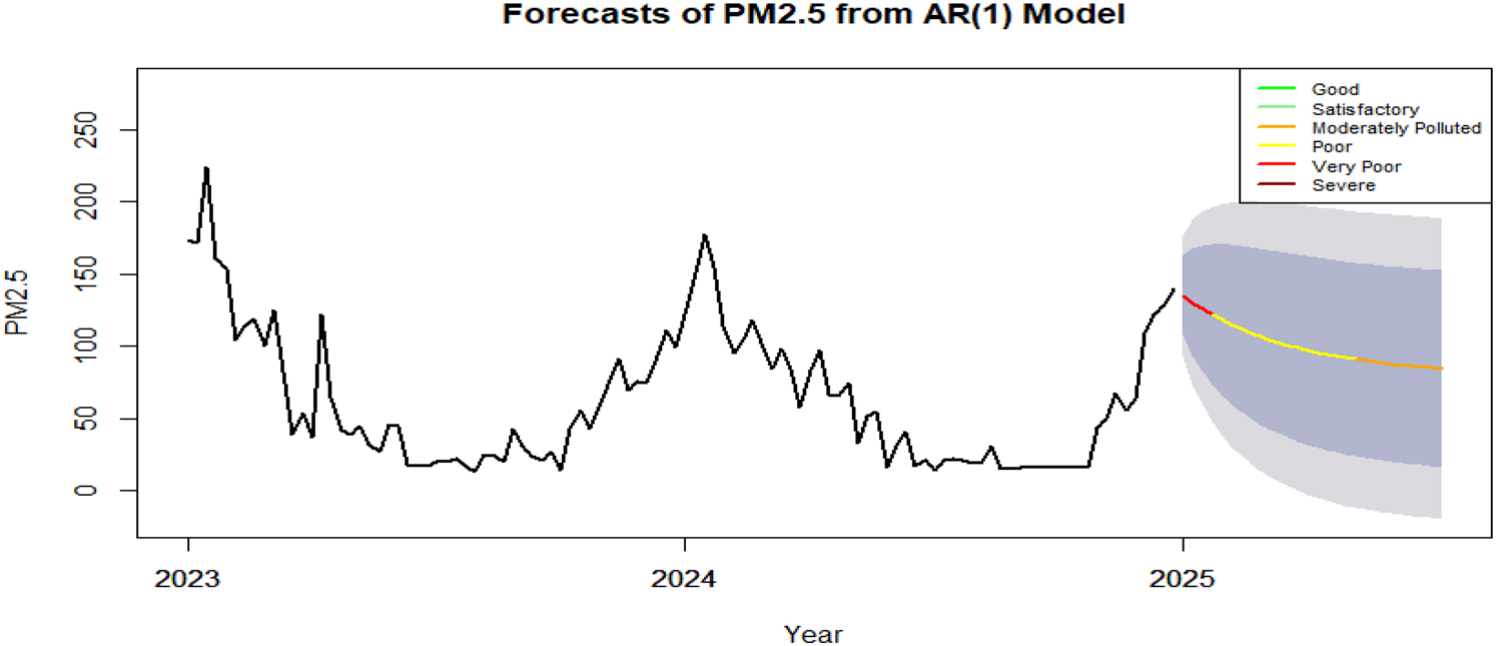
PM_2.5_ level forecast from AR (1) model for the next 28 weeks [Jan -April, 2025].

AR (1) model-based forecast of PM_2.5_ concentrations in Guwahati given in figure 8 provides critical implications for public health and environmental management. The initial forecasted values fall within the ‘Very Poor’ and ‘Poor’ air quality category for the first three months, indicating potentially hazardous conditions for the population. While there is a gradual improvement over time, with PM_2.5_ levels moving from ‘Very Poor’ to ‘Poor’ and eventually to ‘Moderately Polluted’, the overall air quality still remains below acceptable standards during the forecast period. The wider confidence bands observed in the forecast indicate increased variability and uncertainty, suggesting that while the AR(1) model captures the general trend, real-world fluctuations likely due to weather patterns, emissions, and seasonal changes remain difficult to predict precisely. Nevertheless, the model proves effective in identifying broad patterns of air quality deterioration and recovery.

These findings underscore the need for continuous air quality monitoring and proactive policy measures aimed at reducing emissions from key sources such as traffic, industry, and biomass burning. The wider confidence bands indicate greater variation and uncertainty in the forecasted PM_2.5_ levels, which also signals the need for closer attention and monitoring. Moreover, the persistent presence of unhealthy air quality levels reinforces the urgency of implementing evidence-based environmental interventions in Guwahati to protect public health and ensure sustainable urban development.

### Limitations and future directions

The proposed ELRT is developed specifically for detecting AR(1) structures in time series data; however, its performance under model misspecification and non-normally distributed errors has not been evaluated. Future research could explore extending the ELRT framework to accommodate higher-order autoregressive structures such as AR(p) models, adapt it for multivariate time series settings, and develop robust versions capable of addressing model misspecification and non-normal error distributions.

## Conclusion

Identifying a suitable time series model is crucial for obtaining reliable and accurate forecasts. In this study, a distribution-free method, the ELRT is proposed for detecting autoregressive models of order 1. The performance of ELRT is compared with the LB test. The results indicate that ELRT maintains the nominal size and outperforms the Ljung-Box test in terms of power. Real-life application also justifies the suitability of the proposed test for AR (1). The proposed ELRT method is a reliable tool for both researchers and practitioners, excelling in its ability to assess autocorrelation in AR (1) models. This effectiveness enhances the precision and reliability of time series modeling and forecasting, making it a significant contribution to the field. Also, the PM_2.5_ forecasting results underscore the persistent air pollution challenge in Guwahati and highlight the need for more robust strategies to protect public health.

## Data Availability

The dataset utilized in this study are publicly accessible and can be retrieved from the official website of CPCB board of India as: https://airquality.cpcb.gov.in/ccr/#/caaqm-dashboard-all/caaqm-landing/caaqm-comparison-data. After opening the link, the data can be downloaded by selecting State = Assam, City = Guwahati, Station = Railway Colony, Parameter = PM2.5, and the time period from 2022 to 2024.

## List of abbreviations

AR: Autoregressive
AR (1): Autoregressive model of order one
ARIMA: Autoregressive integrated moving average
LB: Ljung-box
ELRT: Empirical likelihood ratio test
OLS: Ordinary least squares
ACF: Autocorrelation function
PACF: Partial autocorrelation function
EL: Empirical likelihood
INAR: Integer-valued autoregressive
